# Mechanisms of light‐induced liposome permeabilization

**DOI:** 10.1002/btm2.10032

**Published:** 2016-09-28

**Authors:** Dyego Miranda, Jonathan F. Lovell

**Affiliations:** ^1^ Dept. of Biomedical Engineering University at Buffalo, State University of New York Buffalo NY 14260

**Keywords:** chemophototherapy, drug delivery, light‐triggered release, liposomes, membrane permeabilization

## Abstract

Liposomes have been widely studied for drug delivery applications. The inclusion of photoactive molecules into liposomes opens the possibility of light‐controlled cargo release to enhance drug biodistribution or bioavailability at target sites. Membrane permeabilization induced by light can be an effective strategy for enhancing cargo delivery with spatial and temporal control, which holds potential for chemophototherapy approaches. Several diverse mechanisms have been reported including light‐induced oxidation, photocrosslinking, photoisomerization, photocleavage, and photothermal release. Here, we review selected recent reports of light‐triggered cargo release from liposomes.

## 1 Introduction

Liposomes have been used extensively for numerous applications, including as drug delivery systems. They have been used as carriers for anti‐cancer chemotherapeutic drugs, as well as for several other indications, such as for treatment of fungal and bacterial infections.^1^ Liposomes can vary in size, ranging from the nano‐ to the micron‐scale, and are generally formed by self‐assembly of amphiphilic lipids in an aqueous solution. Liposomes can load and retain cargo in their hydrophilic core or within the hydrophobic region of the bilayer. This can lead to extended circulation times and improved biodistribution.^2^, ^3^ Adverse side effects from traditional chemotherapies can potentially be reduced in this way.^4^, ^5^ The ability to release a drug, on‐demand, in a controlled and selective manner should increase the efficacy of liposomal therapies by increasing the local concentration or local bioavailability at a specific site of interest. Such approaches make use of internal or external triggers to induce the nanocarrier to release its content whenever and wherever necessary. Several triggers have been demonstrated to release liposomal cargo. A common example of an external trigger is the use of heat to induce membrane permeability,^6^, ^7^, ^8^ and other demonstrated triggers include ultrasound^9^ and magnetic fields.^10^ Alternatively, endogenous triggers at the target sites aim to exploit differential properties of tumors such as lower pH^11^, ^12^, ^13^ and enzymes found in cancer tissues.^14^, ^15^

Light is an intriguing stimulus to remotely trigger cargo release.^16^, ^17^, ^18^, ^19^, ^20^ Chemophototherapy, which has been explored preclinically and clinically, combines chemotherapy and phototherapy and stands to benefit from nanomaterials that release their cargo in response to light.^21^ The advantages of using light as a trigger is that various parameters, such as exposure time, wavelength, beam diameter, and laser intensity can be readily externally modified to adapt to different purposes. Different light wavelengths can be used for laser‐triggered release of cargo. Ultraviolet (UV) and visible light are not ideally suited for treatments that require deep tissue penetration due to high scattering and absorption by tissue components,^22^, ^23^ thus are often restricted to topical applications.^24^, ^25^, ^26^ Conversely, wavelengths in the near‐infrared (NIR; generally 650‐900 nm) can penetrate better into tissues (e.g., up to 1‐2 cm) and are more suitable for light‐triggered cargo release in deeper tissues.^27^, ^28^ Although NIR light penetration is relatively limited in tissues, most areas in the body can be reached with careful treatment planning and with the use of interstitial fibers.

### 1.1 Light‐sensitive liposomes

Liposomes have been studied extensively as biocompatible nanocarriers for a plethora of purposes, including drug and gene delivery, vaccines adjuvants, diagnostic agents, and ingredients for cosmetics and vitamins, among many others.^29^, ^30^ They are generally composed of phospholipids and cholesterol and can be sterically stabilized with polyethylene glycol (PEG) to give “stealth” abilities to delay clearance by the reticuloendothelial system. As liposomes are formed mostly from naturally occurring lipids, they present good biocompatibility and low toxicity. Furthermore, due to their small size and high flexibility, liposomes can passively accumulate in tumor sites via the enhanced permeability and retention effect. Although these features make liposomes an attractive and clinically used drug delivery system, some limitations should still be overcome depending on their formulation; for example, short circulating half‐life, poor entrapment stability, passive leakage and fusion, uptake by undesired organs, and manufacturing‐related problems such as high production costs and time.^30^

Incorporation of photoactive molecules into liposomes has potential to enable light‐triggered cargo release of entrapped molecules. As will be discussed here, several different photoactive molecules are capable of inducing membrane destabilization and permeabilization. Their localization in the liposome is dependent on their intrinsic polarity. Photoactive molecules can also be conjugated to phospholipids, either at the hydrophobic tail or at the head.

Several mechanisms have been reported as a mean for light‐induced membrane destabilization to promote cargo release. These include light‐induced oxidation, photocrosslinking, photoisomerization, photocleavage, and photothermal release. We note that several excellent reviews exploring common mechanisms of cargo release from liposomes, micelles, polymer‐based nanoparticles and other inorganic nanoparticles exist in the literature.^30^, ^31^, ^32^, ^33^, ^34^, ^35^, ^36^

## 2 Mechanisms of light‐triggered release from liposomes

### 2.1 Light‐induced oxidation

One of the most common mechanism used for disruption of nanocarriers is light‐induced oxidation by reactive oxygen species (ROS). ROS have unpaired electrons or unstable bonds. In tissues, high concentrations of ROS leads to several types of damage due to oxidative stress, which includes DNA damage, oxidation of lipids, amino acids and proteins, inflammation, and ultimately, cell apoptosis.^37^, ^38^ Common ROS resulting from chemical reactions include: singlet oxygen (^1^O_2_), hydroxyl radicals (HO•), superoxide (O2•^−^), and hydrogen peroxide (H_2_O_2_).^39^ Photosensitizers (PS) are known to produce singlet oxygen when excited by light at specific wavelength ranges. This mechanism has been extensively explored in photodynamic therapy, in which singlet oxygen derived from excitation of a PS induces oxidative stress, disruption of cellular membranes and cell death.^40^, ^41^, ^42^

Light‐induced generation of singlet oxygen has been used to release cargo from nanoparticles by oxidation of different lipids. The mechanism behind the release is thought to occur via membrane permeabilization caused by oxidization of unsaturated lipids and concomitant formation of pores in the bilayer, which causes the cargo to leak from the nanoparticle, as previously shown by Pashkovskaya et al.^43^ In this study, liposomes formed by unsaturated lipids were shown to release 5,6‐carboxyfluorescein (CF), sulforhodamine B (SRB) and calcein in such manner that the permeability of the dye increased as molecular weight decreased (calcein < SRB < CF).

**Figure 1:**
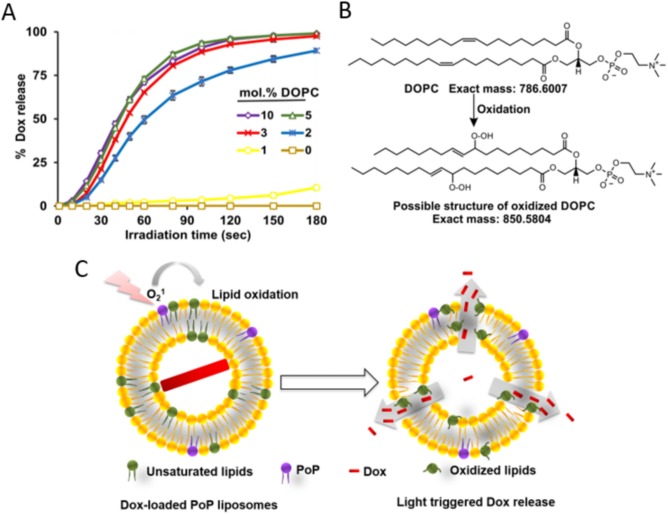
Unsaturated lipids like DOPC accelerate light‐triggered release via lipid oxidation. (A) Dox release at increasing concentrations of unsaturated lipid (DOPC). (B) DOPC oxidation after laser irradiation. (C) Proposed mechanism of light‐induced Dox release with NIR irradiation. Figure used with permission from the publisher^44^

The permeability of the membrane can be influenced by the molar percentage of unsaturated lipids, as shown by Luo et al., where 1,2‐dioleoyl‐sn‐glycero‐3‐phosphocholine (DOPC) was used as a “helper” lipid when combined with 1,2‐distearoyl‐sn‐glycero‐3‐phosphocholine (DSPC).^44^ Increasing DOPC concentration, up to a maximum of 10 mol. %, was found to increase doxorubicin (Dox) release rates when liposomes were permeabilized with NIR at 665 nm (Figure 1A). This study showed that both DOPC and cholesterol are oxidized by singlet oxygen resulting from excitation of porphyrin‐phospholipid (PoP). PoP is a lipid‐like molecule that has a PS (e.g., the chlorophyll‐derived pyropheophorbide‐a (Pyro)) covalently linked to a phospholipid side chain, and can readily be used to form biocompatible and optically active liposomes. PoP is currently being investigated for numerous multimodal imaging and phototherapy purposes.^45^, ^46^, ^47^, ^48^, ^49^, ^50^, ^51^, ^52^ Porphyrins themselves possess intrinsic qualities that make them attractive as theranostic agents.^53^, ^54^ PoP typically comprises a mono‐carboxylic porphyrin derivative esterified to the central sn‐2 hydroxyl of the glycerol backbone of phosphatidylcholine containing a palmitoyl group at the sn‐1 position. To induce Dox release, light‐induced peroxidation by singlet oxygen was suggested to occur mainly on the double bonds present in the two acyl chains of DOPC (Figure 1B), resulting in membrane permeabilization (Figure 1C).^44^ PoP liposomes were able to release variable cargos such as Dox, calcein, SRB, and gentamicin in response to NIR light.^19^

Rwei et al. demonstrated a novel liposome strategy that could be used as an injectable drug delivery system to allow patients to modulate the timing, duration, and intensity of local anesthesia.^55^ A NIR‐absorbing phthalocyanine was entrapped in lipid bilayers containing unsaturated lipids, and the liposomes encapsulated a potent anesthetic agent; tetrodotoxin. Long wavelength light at 730 nm triggered tetrodotoxin release via peroxidation of unsaturated lipids in the bilayer.

### 2.2 Photocrosslinking

The mechanism by which photocrosslinking induces content release typically occurs via polymerization of unsaturated bonds located in the hydrophobic domain of the bilayer. When photosensitive polymerizable moieties are irradiated with a specific wavelength of light, the crosslink reaction between them causes the bilayer to shrink in the surrounding domain where the sensitizers are present. This causes the bilayer natural packing to undergo conformational changes, which in turn correlates with local pore formation, increasing membrane permeability and content leakage.

It has been shown that liposomes composed of a photopolymerizable phospholipid, 1,2 bis(tricosa‐10,12‐diynoyl)‐sn‐glycero‐3‐phosphocholine (DC_8,9_PC), 1,2‐dipalmitoyl‐sn‐glycero‐3‐phosphocholine (DPPC) and DSPE‐PEG2000 at specific molar ratios were able to release calcein and Dox upon light exposure.^56^, ^57^ Treating these liposomes with either 254 nm UV light or 514 nm light resulted in cargo release, although the mechanisms of release was different for both wavelengths. DC_8,9_PC photocrosslinking was observed when the liposomes were treated with 254 nm light, which caused the polymerization and disruption of the liposome membrane. The mechanism behind the light‐triggered release using 514 nm light was thought to be related to the generation of ROS, which was not observed when the liposomes were treated with 254 nm light.

**Figure 2:**
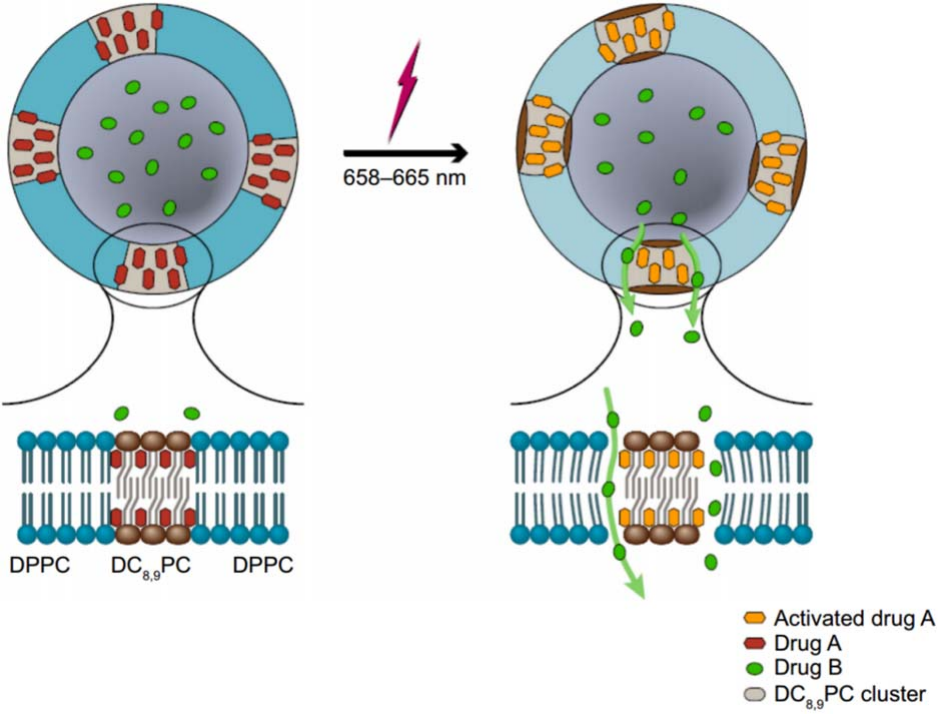
Proposed mechanism of light‐triggered release of calcein from Pocket liposomes. Photo‐activation of HPPH causes the local DC_8,9_PC cluster to form “pockets” in the bilayer, allowing internal cargo to be released. Figure used with permission from the publisher^58^

Recently, the same group introduced a novel light‐triggered release technique using HPPH encapsulated into DPPC:DC_8,9_PC:DSPE‐PEG2000 liposomes.^58^ HPPH co‐encapsulated with the fluorescent dye calcein, and exposure to 660 nm (90 mW) light caused some of the calcein to be released from the liposomes after a 5 min treatment compared with the untreated liposomes. HPPH is thought to be incorporated in the bilayer into “Pockets” around DC_8,9_PC and is excited by wavelengths around 665 nm. The activation of DC_8,9_PC by light was hypothesized to cause the pockets to become unstable, inducing defects on the membrane with concomitant calcein release (Figure 2). The results suggest that calcein was released via a mechanism involving formation of pores in the membrane. In vivo studies with mice bearing an orthotropic breast cancer model of triple negative breast cancer cells expressing luciferase showed that calcein was released in the treated tumor region after laser treatment. The measurement of the fluorescence increase emitted by calcein (due to the loss of its self‐quenching properties) was higher in liposomes containing HPPH and calcein when compared with liposomes encapsulating calcein only, which indicates that only liposomes containing HPPH were capable of light‐triggered release. Tumor regression was also observed in mice treated with HPPH and calcein encapsulated liposomes. After laser treatment and following days after that, tumor bioluminescence signal and volume decreased.

### 2.3 Photoisomerization

**Figure 3:**
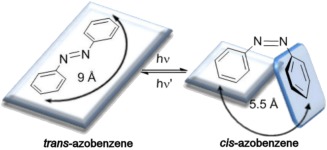
Conformational change of the two phenyl rings of azobenzene. The transition from the trans to cis isomer is normally achieved after light irradiation at wavelengths ranging from 320‐350 nm. This reaction is reversible by irradiation at 400‐450 nm. Figure used with permission from the publisher^62^

Photoisomerization relies on the propensity for certain molecules to undergo a conformational change upon light stimulation. In those molecules, often based on azobenzenes, isomerization occurs when their spatial orientation switches from the trans to cis state. When incorporated into liposomal membranes, for example, this transition is associated with bilayer disruption followed by cargo release.^59^, ^60^ Azobenzenes have two phenyl rings which are interconnected by two nitrogen atoms paired by a double bond. Upon light exposure, the double bond between the nitrogen atoms undergoes an angular conformation change, which leads the molecule changing from having a more apolar (trans) conformation to a more polar (cis) one (Figure 3).^61^, ^62^

Azobenzenes are, in most cases, sensitive to light in the UV spectra, although their activation has also been reported with wavelengths in the range of 530‐560 nm.^63^ The transition from trans to cis can be triggered by light irradiation at wavelengths ranging from 320‐350 nm and the reverse reaction can be triggered by irradiation at 400‐450 nm. The reversible reaction can also be achieved by heat increase, although this process is slower than the photochemical counterpart.^62^ This reversible transition is important, because it enables nanocarriers to be more dynamic by allowing, on‐demand, external control of cargo release through changes in membrane permeability. The reverse switch from cis to trans state decreases membrane permeability, “locking” the nanocarrier once again.

**Figure 4:**
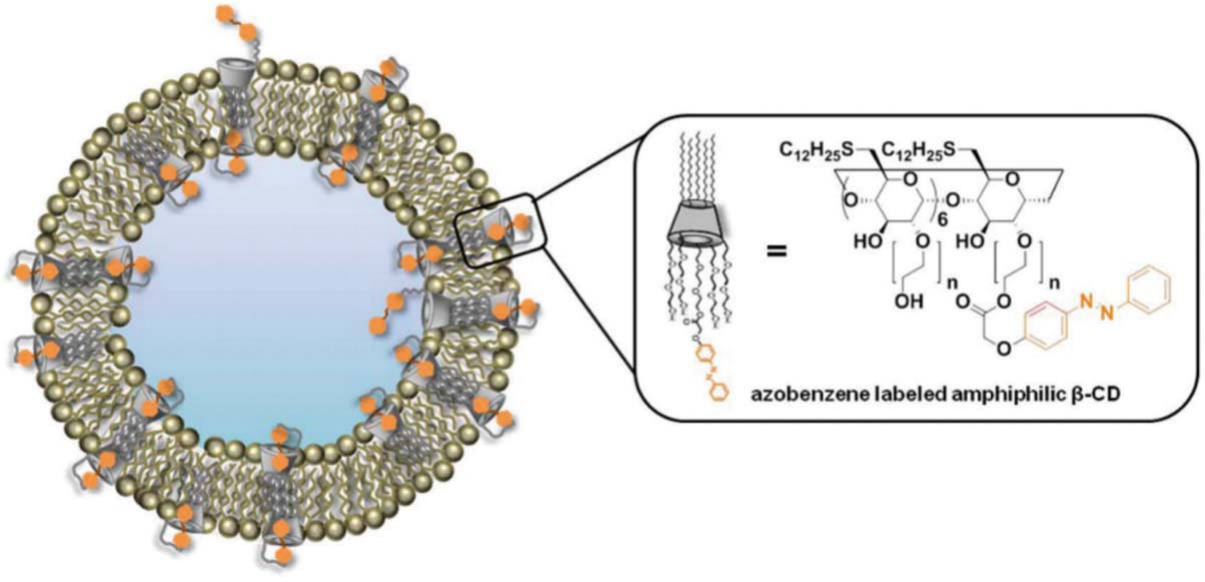
A strategy for the use of azobenzene moieties to control cargo release from liposomes. In this example, azobenzene moieties are covalently attached to β‐cyclodextrin to act as “host‐guest” molecules. After light irradiation, the transition from the trans to cis state leads azobenzene to leave the transmembrane cavity due to its larger size when compared with the trans isomer. Figure used with permission from the publisher^64^

Azobenzene moieties were also reported to act as “host‐guest” molecules when covalently attached to amphiphilic β‐cyclodextrin (Azo‐CD).^64^ The incorporation of transmembrane Azo‐CD within the bilayer (Figure 4) generates a system that can control the release of SRB upon UV light irradiation (λ = 350 nm). When in the trans state, the azobenzene moiety fits inside the β‐cyclodextrin cavity, but when irradiated with UV light, the switch to the cis state causes the polar isomer to move out of the cavity, allowing the release of SRB. The use of azobenzene as photoswitchable transmembrane ion channel was also recently demonstrated by Yang et al.^65^

Azobenzene photoisomerization and cargo release were also achieved using non‐phospholipid liposomes, as reported by Cui et al.^66^ Photosensitive liposomes composed of decyl‐azobenzyl‐triethylammonium [AzoC_10_N^+^] and cholesterol sulfate (Schol) presented low passive permeability but still retained phototriggering characteristics. Self‐assembling of the trans form of AzoC_10_N^+^ with a high proportion of Schol generates a tightly packed bilayer. Upon 350 nm light irradiation, the cis form induces the formation of defects into the membranes followed by solute (SRB) release.

In general, azobenzene photoisomerization is a well‐known mechanism and the attachment of azobenzene moieties to phospholipids has been explored.^60^ The versatility for its use and the reversible character of the reaction can be explored in different manners, which makes it a promising candidate to be used as a light‐triggered mechanism.

### 2.4 Photocleavage

The general mechanism of photocleavage involves the incorporation of a photolabile group, often *o*‐nitrobenzyl (2‐nitrobenzyl),^67^ into a lipid, which can be cleaved upon light irradiation with wavelengths in the UV/visible region, generally in the range of 320‐400 nm. Hydrolysis of *o*‐nitrobenzyl results in the separation of hydrophilic and hydrophobic groups from the amphiphilic phospholipid, which causes membrane destabilization and subsequent cargo release. Chandra et al. reported photocleavable liposomes.^68^, ^69^ In their work, *o*‐nitrobenzyl was used as a linker to conjugate the alkyl chain of stearyl amine to polar head groups with negative charge due to the incorporation of charged amino acids. Liposomes formed from DSPC (95%) and photocleavable lipid (5%) released CF upon 365 nm light irradiation.

**Figure 5:**
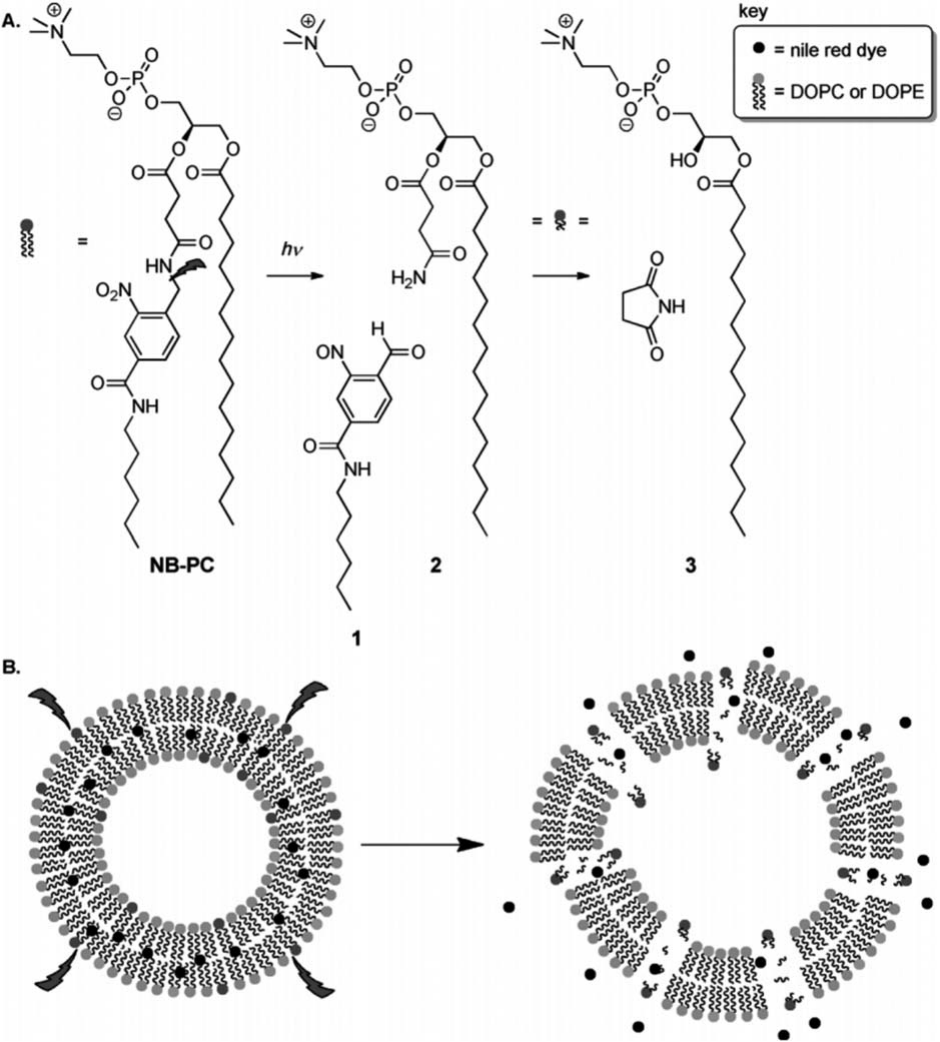
Proposed photocleavage mechanism for NB‐PC and drug release. (A) During irradiation, the C‐N bond in the linker is cleaved, producing aldehyde (1) and amide (2). Afterwards, intramolecular cyclization could also generate succinimide and LPC (3). (B) Nile red is released from the bilayer after NB‐PC photolysis. Figure used with permission from the publisher^70^

Bayer et al. reported the synthesis of a new photocleavable phospholipid, namely NB‐PC.^70^ Stable NB‐PC liposomes were generated from the modification of the natural occurring phosphatidylcholine (PC) to include 2‐nitrobenzyl in the acyl chain at the *sn*‐2 position. The natural PC head group was kept intact to keep the photocleavable lipid more similar to their natural counterpart, since PC is a major component of cell membranes. The resulting phospholipid, was evaluated when liposomes were prepared with different concentrations of PC, phosphatidylethanolamine (PE), polyethylene glycol‐PE (PEG‐PE) and cholesterol. It was shown that under light irradiation (350 nm) release of nile red increased with increasing concentration of NB‐PC. Due to the resemblance of NB‐PC to natural PC, NB‐PC liposomes present high stability even with increasing concentration of NB‐PC. The mechanism proposed for the photocleavage and membrane disruption is depicted in Figure 5.

Phospholipid photocleavage can be achieved using molecules other than nitrobenzyl derivatives, as demonstrated by Wan et al., by using synthetic phosphatidylcholine‐like lipids in which a photolabile dithiane‐based tether served as a linker between the hydrophobic tails and the phosphocholine headgroups.^71^ Liposomes were composed of egg palmitoyl‐oleoyl phosphatidylcholine, cholesterol, and the photolabile lipid with entrapped pyridine as a probe for detection. Irradiation with 300 nm light for 75 min decreased the leakage lifetime from 20 hr (in the dark) to 1.5 hrs. NMR analysis also showed that the light treatment could cleave about 60% of the photolabile lipid.

### 2.5 Photothermal release

**Figure 6:**
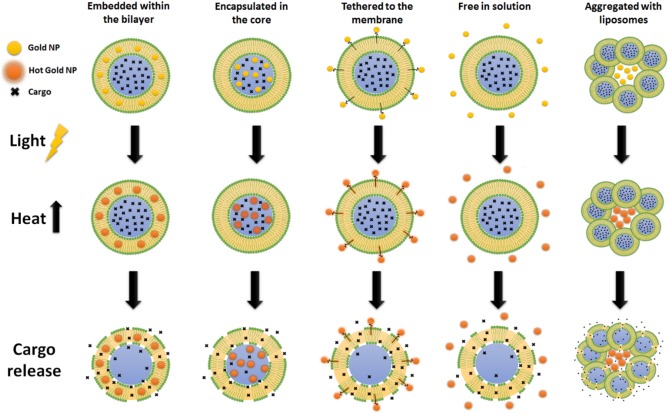
Photothermal release from liposomes using gold nanoparticles (NP). Membrane disruption can be triggered with the gold NP located at different positions such as embedded within the bilayer, encapsulated in the core, tethered to the membrane, free in solution or aggregated with liposomes

Photothermal approaches are based on the conversion of light into heat to induce liposome permeabilization. Several materials can be used as photothermal transducers to trigger on demand drug delivery. For instance, gold nanoparticles (AuNPs) can be used to enhance light‐triggered release due to their suitability for photothermal conversion based on surface plasmon resonance and hot electron mechanisms. AuNPs are able to rapidly and efficiently absorb visible, UV^72^ and NIR light^73^ and release energy as heat on the scale of picoseconds.^74^ This effect is enhanced especially if the light wavelength matches AuNP absorption bands. This phenomenon generates a heated electron gas that rapidly exchanges energy with the particle structure, which then, dissipates this energy in the surrounding medium.^36^, ^75^ When proximal to liposomes, the high temperatures achieved by AuNPs can induce membrane stress and rupture, followed by cargo release. In some cases, instead of membrane rupture, the photothermal effect induces a phase transition in the bilayer, which makes it leakier and leads to an increased cargo release.^72^ The location of AuNPs or any other light absorber is variable and dependent on its hydrophobicity and charge. They can be encapsulated within the liposomal core,^76^, ^77^ tethered to the membrane,^73^, ^77^, ^78^ inserted within the bilayer,^72^ free in liposome solution^77^, ^79^ or assembled as aggregates with liposomes (Figure 6).^80^

Thermosensitive liposomes (TSL) can be activated with photothermal transducers and NIR light is an appealing wavelength for clinical use due to its deeper tissue penetrance. Kwon et al. developed a TSL system tethering gold clusters on liposome bilayers with NIR sensitivity.^73^ Light absorbed by the gold cluster is converted into heat and transferred to the TSL, causing membrane destabilization. The system was effective in releasing Dox and decreasing cell viability and promoting antitumor effect. TSLs have also been shown to release Dox following photothermal activation from non‐tethered nanorods located in the tumor vicinity.^81^ Another approach coupled gold nanorods (GNRs) with TSLs containing tetrodotoxin to use NIR light to repeatedly trigger (over multiple days) local nerve blocking on demand in a rat model.^82^

Photothermal release does not need be related to whole sample temperature increase to promote liposomal disruption. As demonstrated by Wu et al., membrane permeabilization was induced by a “sonication‐like” process in the presence of hollow gold nanoshells (HGNs).^78^ In this process, irradiation of pulsed NIR laser was shown to generate microbubble cavitation, with increasing intensity as HGNs became closer to the liposomal membrane.^77^, ^78^, ^83^ For example, free HGNs in solution induced a maximum of 35% release of CF, while HGNs tethered to the membrane via a thiol‐PEG‐lipid linker increased CF release to 93%. When HGNs reach high temperatures, they induce the formation of unstable vapor microbubbles, which grow rapidly and then collapse, inducing membrane permeabilization. This mechanism appears similar to the transient cavitation effect induced by ultrasound.^84^ Morphology analysis of the liposomes showed that the hollow structure of HGNs collapsed due the melting process. Interestingly, although HGNs are heated above their melting point, the total increase in the sample temperature was less than 1°C.^78^ This approach has been advanced by incorporation of lysolipids to preformed liposomes to create sterically stable, TSLs that release contents under NIR light.^85^ In another study, Mathiyazhakan et al.^86^ reported that photoresponsive liposomes loaded with AuNPs failed to release calcein upon 514 nm continuous‐wave laser excitation (with an approximate power density of 12 mW cm^−2^), but successfully released it with an Nd:YAG pulse laser (pulse duration of 6 ns) which was able to instantaneously deliver a power density of 167 kW/cm^2^ per pulse. At the pulse frequency fixed at 1 Hz, the calculated average energy delivered to the liposomes was only 1 mJ/cm^2^, which is lower than that delivered by the continuous‐wave laser. The mechanism of release was thought to be triggered by microbubble cavitation generated by the high power density delivered in each laser pulse.

Beyond AuNPs, other light‐absorbing agents that are incorporated into TSL have been shown to enhance photothermal release. Viitala et al. used both GNRs encapsulated within the liposome core and indocyanine green (ICG) within the bilayer. ICG absorbs light in the NIR spectrum and by changing the aspect ratio of GNRs length, they could further absorb NIR light as well.^76^ When the light power density required to cause a phase transition in the lipid bilayer was applied (at least 18 W cm^−2^), liposomes encapsulating GNRs released approximately 57% of calcein, while almost 80% was released from liposomes embedded with ICG. The explanation for the higher release of calcein using ICG, is that ICG remains within the bilayer. Due to this close proximity, it is able to cause higher membrane destabilization when compared with GNRs located in the core. The more energy that is delivered, the more physical deformations occur on the membrane that, eventually, breaks apart and releases its content. The approach of using ICG as a photothermal transducer was extended for triggered release of entrapped model drug cargos.^87^

## 3 Conclusion

Light‐triggered cargo release from liposomes involves membrane destabilization and permeabilization. In light‐induced lipid oxidation, unsaturated phospholipids are oxidized by ROS such as singlet oxygen. Photocrosslinking occurs via polymerization of photopolymerizable lipids and subsequent membrane permeabilization. The mechanism of photoisomerization‐triggered release is related to molecular conformational changes (frequently involving azobenzenes) induced by light irradiation. In photocleavage mechanisms, the incorporation of a photolabile group (e.g., *o*‐nitrobenzyl) onto a lipid makes it susceptible to hydrolysis upon light irradiation. In photothermal release, a photothermal agent converts light into heat which is transferred to the bilayer to induce membrane permeability.

Although light‐triggered drug release from liposomes and other nanoparticles has been the focus of extensive academic interest, clinical translation has been limited, despite the novelty of these materials. As a local therapeutic modality, these approaches must address clinical needs that are currently not satisfactorily met by surgery, radiation therapy, or other locally acting techniques, that often do not require systemic administration of potentially hazardous materials. In addition, limited depth penetration of light in biological tissues complicates treatment of larger tumors. Using wavelengths in the NIR can partially address this, but penetration depth remains just a couple of cm. On the other hand, with proper treatment planning, fiber optic design and interstitial light delivery, delivery of light to compact, well‐defined volumes holds potential to precisely ablate target tissues while sparing healthy ones. Alternatively, selecting relatively superficial disease indications in which deep light penetration is not required could be a good strategy to more rapidly translate light‐activated drug release technologies. For example, Visudyne, a lipid formulation of Verteporfin (a hydrophobic chlorin PS), is a successful example of a light‐activated agent for local treatment of blood vessel disorders in the eye, where light penetration is less challenging.

In conclusion, multiple mechanisms for light‐triggered cargo release from liposomes have been developed and demonstrated as strategies with potential for improved localized drug delivery. For translation of light‐triggered liposomes, additional efforts are required to study local and systemic toxicity of the photoactive components, test efficacy in large animal models and eventually move into human clinical trials.
